# The Entourage Effect in Cannabis Medicinal Products: A Comprehensive Review

**DOI:** 10.3390/ph17111543

**Published:** 2024-11-17

**Authors:** Rebeca André, Ana Patrícia Gomes, Catarina Pereira-Leite, António Marques-da-Costa, Luis Monteiro Rodrigues, Michael Sassano, Patricia Rijo, Maria do Céu Costa

**Affiliations:** 1 Escola de Ciências e Tecnologias da Saúde (ECTS), CBIOS—Universidade Lusófona’s Research Center for Biosciences & Health Technologies, Campo Grande 376, 1749-024 Lisboa, Portugal; rebeca.andre@ulusofona.pt (R.A.); pg@somaipharma.eu (A.P.G.); catarina.leite@ulusofona.pt (C.P.-L.); monteiro.rodrigues@ulusofona.pt (L.M.R.); 2 SOMAÍ Pharmaceuticals, R. 13 de Maio 52, 2580-507 Carregado, Portugal; amc@somaipharma.eu (A.M.-d.-C.);; 3 Laboratório Associado para a Química Verde, REQUIMTE, Departamento de Ciências Químicas, Faculdade de Farmácia, Universidade do Porto, Rua de Jorge Viterbo Ferreira 228, 4050-313 Porto, Portugal; 4 Instituto de Investigação do Medicamento (iMed.ULisboa), Faculdade de Farmácia, Universidade de Lisboa, 1649-003 Lisboa, Portugal; 5 NICiTeS, Polytechnic Institute of Lusophony, ERISA-Escola Superior de Saúde Ribeiro Sanches, Rua do Telhal aos Olivais 8, 1950-396 Lisboa, Portugal

**Keywords:** cannabis, cannabinoids, terpene(s), entourage effect, influencers

## Abstract

This study explores the complementary or synergistic effects of medicinal cannabis constituents, particularly terpenes, concerning their therapeutic potential, known as the entourage effect. A systematic review of the literature on cannabis “entourage effects” was conducted using the PRISMA model. Two research questions directed the review: (1) What are the physiological effects of terpenes and terpenoids found in cannabis? (2) What are the proven “entourage effects” of terpenes in cannabis? The initial approach involved an exploratory search in electronic databases using predefined keywords and Boolean phrases across PubMed/MEDLINE, Web of Science, and EBSCO databases using Medical Subject Headings (MeSH). Analysis of published studies shows no evidence of neuroprotective or anti-aggregatory effects of *α*-pinene and *β*-pinene against *β*-amyloid-mediated toxicity; however, modest lipid peroxidation inhibition by *α*-pinene, *β* pinene, and terpinolene may contribute to the multifaceted neuroprotection properties of these *C. sativa* L. prevalent monoterpenes and the triterpene friedelin. Myrcene demonstrated anti-inflammatory proprieties topically; however, in combination with CBD, it did not show significant additional differences. Exploratory evidence suggests various therapeutic benefits of terpenes, such as myrcene for relaxation; linalool as a sleep aid and to relieve exhaustion and mental stress; D-limonene as an analgesic; caryophyllene for cold tolerance and analgesia; valencene for cartilage protection; borneol for antinociceptive and anticonvulsant potential; and eucalyptol for muscle pain. While exploratory research suggests terpenes as influencers in the therapeutic benefits of cannabinoids, the potential for synergistic or additive enhancement of cannabinoid efficacy by terpenes remains unproven. Further clinical trials are needed to confirm any terpenes “entourage effects.”

## 1. Introduction

### 1.1. Entourage Effects: Concepts and Evolution

The entourage effect refers to the idea that compounds found in cannabis—such as cannabinoids, terpenes, and flavonoids—can work synergistically, enhancing the therapeutic effect outcome compared to the effects when they are used individually.

**Figure 1:**
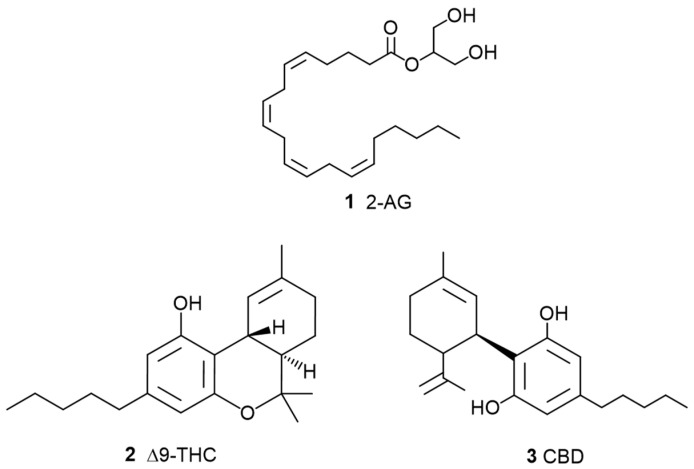
Chemical structures of the key cannabinoids related to the entourage effect, 2-Arachidonoylglycerol (2-AG, **1**), Δ9-tetrahydrocannabinol (THC, **2**), and cannabidiol (CBD, **3**).

The term entourage effect was first introduced by Ben-Shabat et al., 1998 [1], in a study exploring the interaction of endogenous cannabis compounds, where it was found that inactive metabolites, such as fatty acid glycerol esters, could enhance the effects of the endocannabinoid 2-Arachidonoylglycerol (2-AG) (**1**, Figure 1) when combined in both in vitro and in vivo studies. The enhanced effect observed within specific metabolite concentration ranges was described as the entourage effect and suggests a potential role in the therapeutic application of cannabis-based products. The authors discussed that bioactive compounds from plants are often accompanied by chemically related substances, referred to as entourage compounds, which may appear inactive when administered individually.

A more recent scoping review by Christensen et al., 2023 [2], elaborates on the entourage effect using traditional pharmacological concepts like those applied in other plant-based medicinal products and multi-drug interactions, such as synergy and bio-enhancement. The idea was later compared to polypharmacy, particularly in full-spectrum medicinal cannabis products, which are believed to produce a stronger effect compared to isolated compounds such as Δ9-tetrahydrocannabinol (THC) (**2**, Figure 1) and cannabidiol (CBD) (**3**, Figure 1), as well as their synthetic analogues. Proponents argue that the entourage effect explains why many patients report better results with full-spectrum cannabis products [3]. However, despite these observations and since its introduction, the pharmacological basis and relevance of the entourage effect have been debated, with critics asserting that the term lacks scientific support and is primarily used as a marketing tool in the cannabis industry.

Therefore, the entourage effect is closely tied to the diversity of compounds found in cannabis plants, and research has increasingly focused on exploring and understanding the pharmacological effects underlying it [4]. While the exact mechanisms of action remain unclear, it is widely believed that they involve interactions between compounds in winterized extract, where cannabinoids and other compounds like terpenes and flavonoids are preserved while plant waxes are removed. These interactions occur when one compound’s effect is either enhanced or diminished by the presence of others [5,6,7]. The entourage effect is often attributed to beneficial synergistic effects, with discussions typically avoiding potential antagonistic or additive adverse effects [8].

Two distinct types of entourage effects have been defined for cannabis-derived compounds: intra-entourage, which involves interactions between cannabinoids or terpenes, and inter-entourage, which refers to interactions between cannabinoids and terpenes [9].

A wide range of chemical classes—18 in total—including terpenes, nitrogenous compounds, hydrocarbons, fatty acids, and amino acids, all contribute to the pharmacological and toxicological properties of cannabis [10].

**Figure 2:**
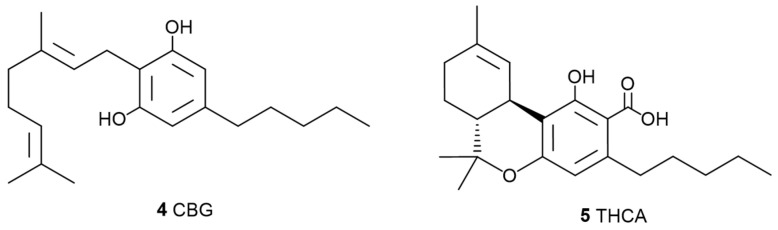
Chemical structures of cannabigerol (CBG, **4**) and tetrahydrocannabinolic acid (THCA, **5**), minor cannabinoids with possible therapeutic potential.

Botanical synergy in cannabis was first demonstrated with THC (**2**) and other “minor” cannabinoids. In a study by Johnson et al., 2010, a cannabis-based extract tested on patients with intractable pain found that a THC (**2**)-dominant extract showed no significant improvement over a placebo, whereas a whole plant extract that included CBD (**3**) demonstrated a considerable improvement in pain relief [11]. Similarly, animal studies have shown that full-spectrum cannabis extract produced a stronger analgesic effect than treatments solely with pure cannabinoids (**3**) [12]. Further research has demonstrated synergistic interactions on colorectal cancer cell lines [13]. Different studies using a mouse model for seizures tested different cannabis strains containing an equivalent CBD (**3**) concentration, revealing notable differences in efficacy between the strains. A profiling study of 94 phytocannabinoids across 36 widely used cannabis plants concluded that minor cannabinoids have an impact on the overall efficacy of cannabis plant extracts [14]. Additionally, an in vitro study [15] on breast cancer cell lines found that whole cannabis extracts were more effective than THC (**2**) alone, with increased activity attributed to the presence of “minor” cannabinoids like cannabigerol (CBG) (**4**, Figure 2) and tetrahydrocannabinolic acid (THCA) (**5**, Figure 2).

Phytocannabinoids possess a high antioxidant capacity due to their terpene-phenolic chemical structures with hydroxyl groups. Combined with their lipophilic nature and anti-inflammatory effects, these characteristics further enhance their potential as therapeutic candidates for various systemic disorders. Within the central nervous system (CNS), they can effectively cross the blood–brain barrier (BBB), modulate the immune response, and impact multiple aspects of neurodegenerative processes [16,17]. While these characteristics have been well established for major cannabinoids such as **2** and **3**, minor cannabinoids are still poorly studied. The development of this knowledge will be possible only with a full understanding of the therapeutic potential of *Cannabis sativa*.

**Figure 3:**
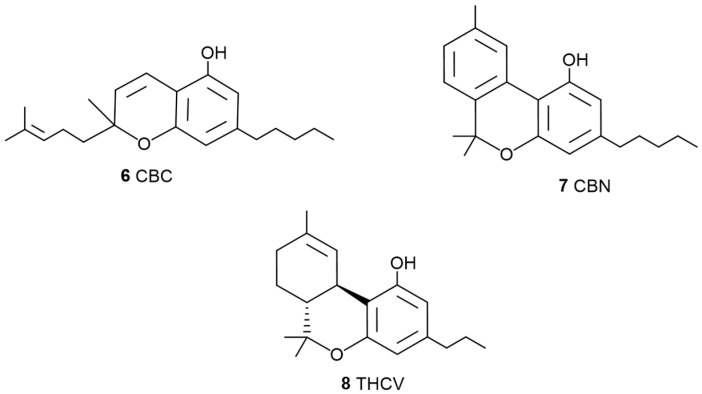
Chemical structures of minor cannabinoids with therapeutic applications, cannabichromene (CBC, **6**), cannabinol (CBN, **7**), and tetrahydrocannabivarin (THCV, **8**). Source: [3].

The scientific community has placed particular emphasis on all cannabinoids that, when isolated, exhibit possible medicinal properties, including not only THC (**2**, Figure 1) but also other cannabinoids such as CBD (**3**, Figure 1), CBG (**4**, Figure 2), cannabichromene (CBC) (**6**, Figure 3), cannabinol (CBN) (**7**, Figure 3), and tetrahydrocannabivarin (THCV) (**8**, Figure 3).

**Figure 4:**
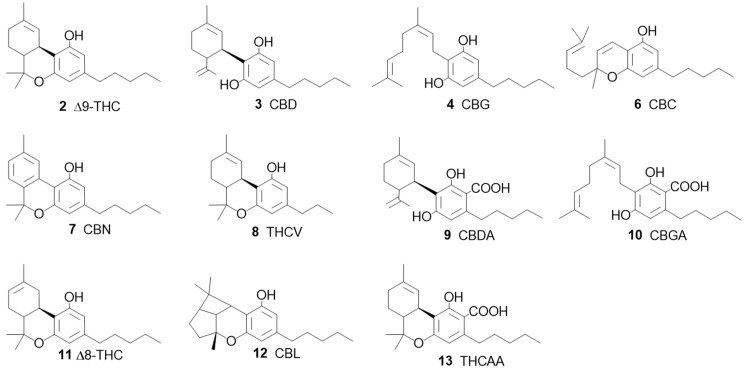
Cannabinoids identified in a single HPLC run: Δ9-tetrahydrocannabinol (Δ9-THC, **2**), cannabidiol (CBD, **3**), cannabigerol (CBG, **4**), cannabichromene (CBC, **6**), cannabinol (CBN, **7**), tetrahydrocannabivarin (THCV, **8**), cannabidiolic acid (CBDA, **9**), cannabigerolic acid (CBGA, **10**), Δ8-tetrahydrocannabinol (Δ8-THC, **11**), cannabicyclol (CBL, **12**), and tetrahydrocannabinolic acid A (THCAA, **13**) [3].

Of particular note are the recent advancements in analytical techniques, such as HPLC, which currently allows the quantification of nearly 100 cannabinoids in a single run, a major evolution since the pioneering work of Wang et al. [18], which initially focused on analyzing 11 cannabinoids (Figure 4). In their study, optimized extraction solvents and a validated UHPLC–UV–MS method were applied to analyze 32 cannabis samples including flowers, leaves, and hashish. These advancements were crucial for elucidating the complex interactions among cannabinoids and understanding the therapeutic potential of cannabis, thereby supporting the concept of the entourage effect.

### 1.2. Cannabis Strains, Genetic Variety, and the Role of Minor Cannabinoids

*Cannabis sativa* L., commonly referred to as “cultivated cannabis”, was likely first described by Classen et al., 2001 [19], and the Latin binominal term was formally adopted by Linnaeus in his comprehensive work of “Species Plantarum” in 1753 [20], where he described it as European hemp. Approximately thirty years later, Lamarck identified a distinct species, *Cannabis indica*, characterized by its bushier form and shorter stature with narrower leaflets, originating from the subcontinent [21]. Since then, the classification of *Cannabis* species has been a topic of ongoing debate [22]. In 1974, Richard Schultes also identified plants from Afghanistan with compact growth and wide leaflets, which he classified as *C. indica* [23]. Meanwhile, other experts, including Ernest Small, argued for a unified classification of species [24]. This argument assumes practical clinical implications in contemporary times, as commercial labels like “sativa” or “indica” are often used to describe the different effects of cannabis varieties when advising patients on which variety to select for their treatment, with effects described as “head up” or “body up”. Therefore, precise definitions became critical in the case of cannabis species.

Aside from species controversy, distinguishing cannabis plants based on their genetic or biochemical characteristics poses a challenge. Terms like “strains” are frequently used inaccurately in commerce, with “variety” or “cultivar” being preferred by botanists to reflect their genetic variants being developed through natural or selective breeding [25,26,27]. However, due to international plant nomenclature rules, cannabis varieties as cultivars must be officially registered, which has been limited to a few examples given the plant’s illegality in many regions [28]. Consequently, some recommend using the term “chemovars” to describe *Cannabis* varieties, which highlights the unique biochemical and genetic attributes of each variant.

Genetic variability can be characterized across the three main species: *Cannabis indica*, *Cannabis ruderalis*, and *Cannabis sativa* [27]. However, polyhybrids have been developed between these species, with varying proportions worldwide, and are commonly marketed as “*Cannabis sativa*”. Each species has a vast array of cultivars and “strains”, with specific genetic profiles that determine variations in the cannabinoid content (e.g., THC and CBD levels), terpene profiles, and plant morphology, ultimately influencing their effects and uses.

Genetic differences have been registered in genetic databanks after breeding and genetic modification programs, with breeding efforts being focused on enhancing terpene profiles and cannabinoid content, with higher resistance to diseases and pests. In parallel, phenotypic variations have been associated with each strain/cultivar. Even within a single cannabis strain, there can be phenotypic variation due to environmental factors, such as soil composition, climate, and cultivation methods. This can lead to differences in plant size, cannabinoid content, and overall quality.

**Figure 5:**
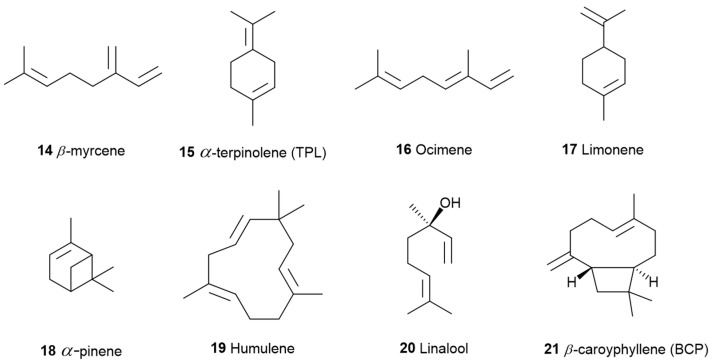
Terpene compounds found in cannabis are considered to qualify as belonging to “Super Classes”.

Coherently, for medicinal use, the European Pharmacopoeia established categories of cannabis based on cannabinoid composition. Type I is characterized by a predominance of THC (**2**), which is commonly available in both medical and recreational marketplaces. Type II refers to cannabis that contains levels of both THC (**2**) and CBD (**3**), while Type III is predominant in CBD (**3**). According to Lewis et al., 2018 [29], high-THC (**2**, Figure 1) and high-myrcene (**14**, Figure 5) chemovars dominate the market, though these profiles may not be suitable for all patients, as some require different biochemical compositions for effective symptom management. Furthermore, this study also found that Type II and III cannabis chemovars, which display higher levels of CBD (**3**) and terpenoids, could offer the potential to enhance THC (**2**) therapeutic effects while minimizing associated adverse effects [29].

Interestingly, in addition to the already identified phytocannabinoids, over the years, researchers have determined that terpenoid content, rather than cannabinoid ratios, is the most distinctive marker between different chemovars [30,31]. Terpenoids, which are mainly produced in glandular trichomes on the unfertilized female flowering tops, are being increasingly recognized for their pharmacological importance. To date, approximately 200 different terpenoids have been isolated in cannabis, with their compositions primarily influenced by genetics rather than environmental factors. Despite their relatively low concentration in cannabis preparations, terpenoids are highly potent and can significantly impact behaviors, modulating activity levels in rodents even when serum levels are minimal or undetectable [32].

Historically, terpenoid concentrations in cannabis flowers were approximately 1%, with up to 10% found in trichomes, but selective breeding has led to terpenoid flower concentrations exceeding 3.5%. The pharmacological effects and ecological roles of terpenoids, which contribute to the synergistic properties and therefore to the entourage effects of cannabis, have been thoroughly explored in the literature [33,34,35], and eight predominate forms the Terpene Super Classes: myrcene (**14**), terpinolene (**15**), ocimene (**16**), limonene (**17**), *α*-pinene (**18**), humulene (**19**), linalool (**20**), and *β*-caryophyllene (**21**) (Figure 5).

Advances in molecular biology have furthered the understanding of cannabis genetics. In 2020, Zhang et al., 2020 [36], estimated the population structure and the genetic diversity of cannabis using the 59 (72 loci) validated polymorphic simple sequence repeat markers (SSRs) and three phenotypic markers. Their genome-wide analyses offered a basis for deeper investigations into cannabis species through techniques such as molecular-assisted breeding, association analysis, and quantitative trait loci (QTL) mapping. These studies, combined with the exchange of cannabis germplasms between regions, pave the way for the introduction of new cannabis varieties on a global scale.

Molecular markers have also been used to estimate genetic diversity, namely, NMI101, a single-hexanucleotide short tandem repeat (STR), was applied to study 93 processed seeds [37]. An analysis utilizing single-nucleotide polymorphisms (SNPs) in 81 marijuana and 43 hemp samples demonstrated significant genome-wide differences, with hemp showing higher genetic similarity to *Cannabis indica* than to *Cannabis sativa* [38]. Another study classified 115 hemp germplasm resources into four groups using expressed sequence tag SSRs (EST-SSRs) [39]. SSRs have been shown to exhibit high levels of polymorphism, with genomic SSRs proven to be more stable and polymorphic than EST-SSRs [40]. Several studies have explored cannabis classification, its genealogical classification, and population structure; however, the genetic links between geographically related strains remain unclear, requiring more accurate molecular markers to assess the diversity within the cannabis population.

For example, Ioannidis et al., 2022 [41], assessed the genetic stability of regenerated and micropropagated varieties of *Cannabis sativa* L. plants with high CBD (**3**) and high CBG (**4**) levels using SSRs, suggesting that in vitro propagation protocols are suitable for large-scale cultivation. This research also highlights the importance of understanding the anthropological and genetic variability of cannabis, which is crucial for its responsible cultivation, regulation, and medicinal use [42]. As legal and cultural perspectives on cannabis continue to evolve, so do its use and perception around the world.

### 1.3. The Phytocannabinoid Entourage

THC (**2**) has been identified as a partial agonist of both cannabinoid type 1 receptor (CB1R) and cannabinoid type 2 receptor (CB2R). It also interacts with various other targets, as demonstrated in several pre-clinical studies [2] (Table S1). The effects of THC (**2**) can vary between antagonistic and agonistic depending on factors such as the presence of additional ligands that bind to the same targets (e.g., endocannabinoids or other cannabinoids derived from cannabis), the receptor expression state, and cell type. Additionally, the concentration of compounds co-administered with THC (**2**), such as entourage compounds, can impact its pharmacological effects [43,44].

**Figure 6:**
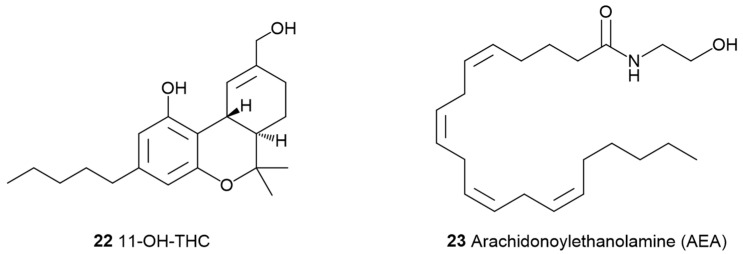
Chemical structures of 11-OH-THC (**22**), a potent metabolite of THC, and arachidonoylethanolamine (AEA, **23**), an endocannabinoid degraded by FAAH.

Cannabinoid **3** (Figure 1) binds with numerous biological targets (summarized in Table S1), giving it a complex and broad pharmacological profile [2,45]. Its polypharmacology is still under extensive investigation for various therapeutic applications, including neuropsychiatric, neurological, and inflammatory disorders [16,46]. Certain mechanisms, like acting as an allosteric negative modulator of CB1 [47], can impact the bioactivity of THC [47]. This has prompted the suggestion that CBD functions as an entourage compound [48]. Additionally, CBD (**3,** Figure 1) can affect the pharmacokinetics of THC (**2**, Figure 1) by inhibiting some hepatic enzymes, such as those in the cytochrome P450 family, slowing the conversion of **2** into its more potent psychoactive metabolite, 11-OH-THC (**22**, Figure 6). CBD (**3**) can also modulate endocannabinoid levels by inhibiting fatty acid amide hydrolase (FAAH), thus inhibiting the degradation of arachidonoylethanolamine (AEA) (**23**, Figure 6) [49].

Different pre-clinical studies covering various diseases have demonstrated that full-spectrum cannabis extracts or combinations of major cannabinoids, with or without other compounds, tend to be more effective than single cannabinoids like THC (**2**) or CBD (**3**) [15,50]. For instance, concerning the anti-cancer potential of cannabis, Blasco-Benito et al., 2018 [15], described a higher anti-tumor effect for a whole plant extract than for pure THC (**2**). This enhanced therapeutic outcome was not attributed to the presence of five commonly found terpenes but rather to the interaction of multiple compounds affecting several targets and mechanisms of action in the extract.

Cannabinoids other than THC (**2**) that are naturally present in cannabis are termed “minor” cannabinoids. Many of the minor cannabinoids display pharmacologic properties that are similar to **2** in that they act as a partial agonist at CB1R and CB2R [51]. To date, studies examining the behavioral pharmacology of minor cannabinoids are limited. Existing preclinical evidence demonstrates that Δ8-tetrahydrocannabinol (Δ8-THC) (**11**) has cannabimimetic effects, producing catalepsy and hypothermia, reducing thermal sensitivity, and altering motor behavior in a dose- and route-dependent manner [52]. Several human studies indicate a weaker potency of Δ8-THC (**11**) at CB1R compared with Δ9-THC (**2**). THCV (**8**), a propyl analog of **2** that exerts biphasic agonist/antagonist action at CB1R and partial agonist action at CB2R, reportedly rescues schizophrenia-like behavior in the phencyclidine rat model of schizophrenia without altering behavior in unmanipulated rats [53].

In a study published in 2019, the authors [54] investigated whether intramuscular injections of three non-psychoactive cannabinoids alone (CBD (**3**), CBC (**6**), CBN (**7**)) and in combination could provide similar relief in sensitized muscle, further minimizing the potential for limiting adverse effects. Although no undesirable effects were found in either treatment group, it was found that the non-psychoactive cannabinoids CBD, CBN, and CBC were less effective than THC at the same concentration (1 mg/mL) in reducing muscle sensitization, a result interpreted according to the fact that these cannabinoids have a weaker binding affinity for CB1R when compared to the affinity of THC: CBN (˜1/10°), CBC (˜1/20°), and CBD (˜1/100°) considering that the same author had demonstrated in a previous study [55] that activation of the CB1R is responsible for the local analgesic effect of THC.

**Figure 7:**
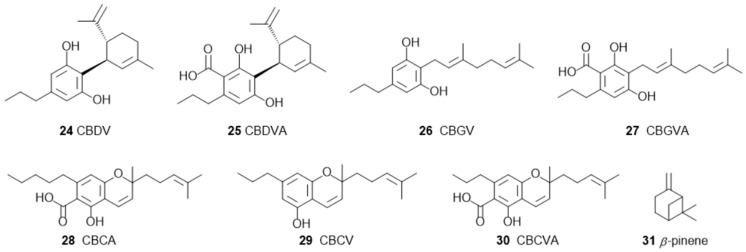
Chemical structures of phytocannabinoids with neuroprotective properties: cannabidivarin (CBDV, **24**), cannabidivarinic acid (CBDVA, **25**), cannabigerivarin (CBGV, **26**), cannabigerovarinic acid (CBGVA, **27**), cannabichromenic acid (CBCA, **28**), cannabichromevarin (CBCV, **29**), and cannabichromevarinic acid (CBCVA, **30**).

A systematic review was published [56] on the neuroprotective properties of phytocannabinoids other than CBD (**3**, Figure 1) and THC (**2**, Figure 1), particularly for the following cannabinoids: CBG (**4**, Figure 2), Δ9-THCA (**5**, Figure 2), CBC (**6**, Figure 3), CBN (**7**, Figure 3), Δ9-THCV (**8**, Figure 3), cannabidiolic acid (CBDA) (**9**, Figure 4), cannabigerolic acid (CBGA) (**10**, Figure 4), cannabidivarin (CBDV) (**24**, Figure 7), cannabidivarinic acid (CBDVA) (**25**, Figure 7), cannabigerivarin (CBGV) (**26**, Figure 7), cannabigerovarinic acid (CBGVA) (**27**, Figure 7), cannabichromenic acid (CBCA) (**28**, Figure 7), cannabichromevarin (CBCV) (**29**, Figure 7), and cannabichromevarinic acid (CBCVA) (**30**, Figure 7). Of 2341 studies, 31 articles met the inclusion criteria. CBG (**4**) (doses ranging from 5 mg.kg^−1^ to 20 mg.kg^−1^) and CBDV (**24**) (doses ranging from 0.2 mg.kg^−1^ to 400 mg.kg^−1^) demonstrated effectiveness in preclinical models of epilepsy and Huntington’s disease. Δ9-THCA (**5**) (20 mg.kg^−1^), CBC (**6**) (10–75 mg.kg^−1^), and Δ9-THCV (**8**) (doses ranging from 0.025 to 2.5 mg.kg^−1^) exhibited potential in hypomobility, seizures, Parkinson’s disease, and Huntington’s disease. Limited mechanistic insights showed that both CBG (**4**) and Δ9-THCA (**5**) d some of their effects via PPARγ receptors. In vivo and in vitro data are included in that review.

Although terpenes have well-identified common targets with cannabinoids [2] (Table S2), separate studies have demonstrated that none of the terpenes, including myrcene (14), limonene (**17**), *α*-pinene (**18**), linalool (**20**), *β*-caryophyllene (**21**) in Figure 5, and *β*-pinene (**31**), were observed to modify potassium channel signaling in AtT20 cells that express CB2 receptors [2]. Additionally, they did not interact with THC at the receptor [57], and they did not influence intracellular calcium levels at the human transient receptor potential ankyrin 1 (hTRPA1) or human transient receptor potential vanilloid 1 (hTRPV1) channel [58].

### 1.4. The Polyphenol Entourage

**Figure 8:**
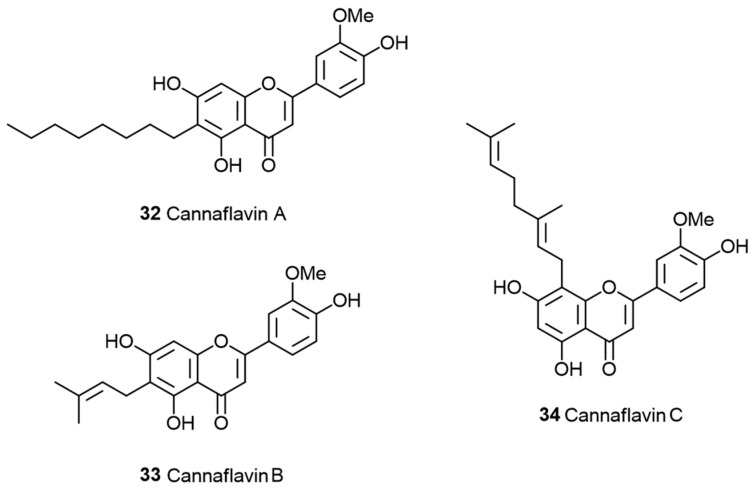
Flavonoids found in cannabis: Cannaflavin A (**32**), cannaflavin B (**33**), and cannaflavin C (**34**) [60].

Similarly to terpenoids and terpenes, cannabis also contains a diverse range of polyphenolic compounds, many of which are prevalent in plants generally. However, some polyphenolics appear to be predominantly or exclusively present in cannabis. Several of these compounds have names starting with the prefix “cann” reflecting their initial identification in cannabis extracts. Unlike terpenes, these polyphenolics are not typically found in trichomes but are usually located in other parts of the plant [59]. This category includes flavonoids with geranyl and prenyl substitutions, such as cannaflavin A (**32**), B (**33**), and C (**34**) (Figure 8), which are found in the flowers, twigs, leaves, and pollen of the cannabis plant. These cannaflavins A–C (**32** to **34**, Figure 8) have been reported to possess neuroprotective, anti-viral, anti-inflammatory, and anti-cancer effects. These compounds are likely to play a role in the overall therapeutic effects of cannabis-derived extracts and may also be considered as entourage compounds.

## 2. Physiological Effects of Terpenes and Terpenoids Found in Cannabis

Terpenes are classified based on the number of carbon atoms they contain, namely, monoterpenes (C_10_), sesquiterpenes (C_15_), diterpenes (C_20_), and triterpenes (C_30_), consisting of two, three, four, or six isoprene units, respectively.

More than 100 different monoterpenes have already been identified in cannabis. This type of terpenes consists of two isoprene units, with a molecular formula C_10_H_16_. Common examples of monoterpenes found in cannabis include myrcene, limonene, pinene, and terpinolene. Each chemovar of cannabis may have a unique terpene profile, which contributes to its distinctive aroma, flavor, and potential therapeutic effects. The exact number and types of monoterpenes can vary across different cannabis varieties.

Sesquiterpenes contain three isoprene units, resulting in a molecular formula that is typically represented as C_15_H_24_. Examples of this type of terpene found in cannabis include *β*-caryophyllene, humulene, and guaiol, among others. Like monoterpenes, sesquiterpenes contribute to the overall aroma, flavor, and potential therapeutic effects of cannabis. The variety of terpenes present in cannabis is one of the factors that contribute to the unique characteristics of different chemovars or “strains”.

**Figure 9:**
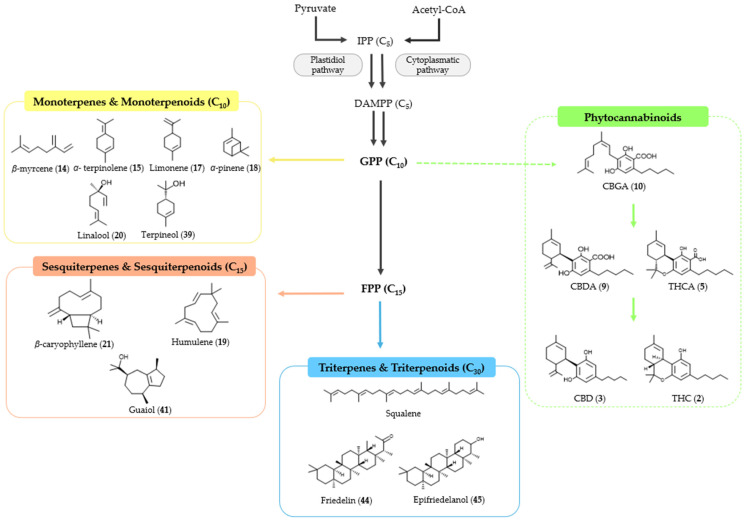
Biosynthesis pathways for cannabinoids and terpenoids, with example molecules from each group.

Additionally, diterpenes consist of four isoprene units corresponding to the molecular formula C_20_H_32_, whereas triterpenes are built from six isoprene units, giving them the molecular formula C_30_H_48_. The linear triterpene squalene backbone, a key component of shark liver oil, is also present in trace amounts in cannabis (Figure 9).

**Figure 10:**
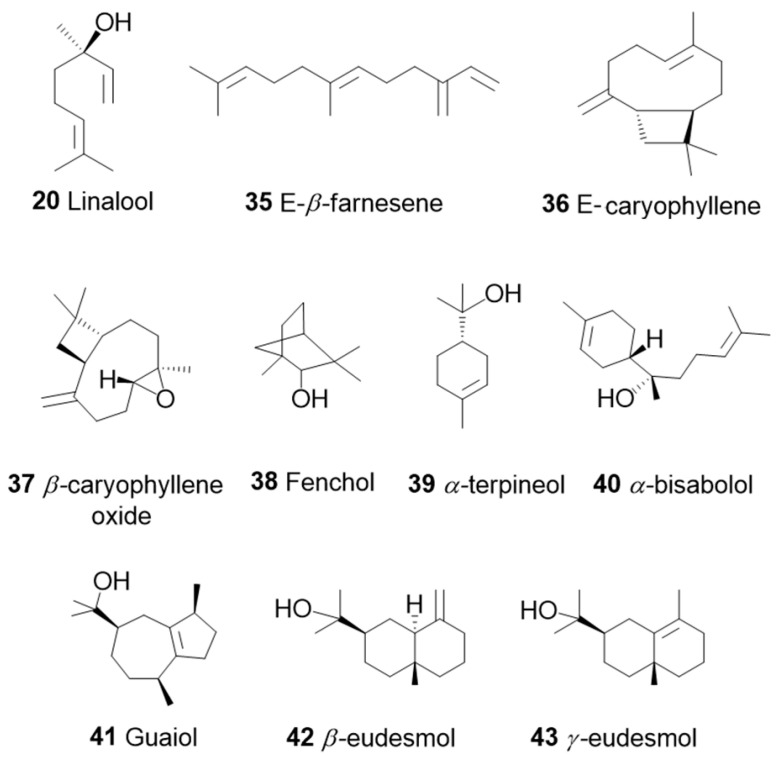
Some mono- and sesqui- terpenoids commonly found in cannabis.

So far, 38 sesquiterpenes and 58 monoterpenes have been characterized across different cannabis genotypes [61,62,63]. The key monoterpene components found were *β*-myrcene (**14**), limonene (**17**), *α*-pinene (**18**), and linalool (**20**), with trace amounts of terpinolene (**15**) and ocimene (**16**) [64], with *β*-caryophyllene (**21**), E-*β*-farnesene (**35**), E-caryophyllene (**36**), and caryophyllene oxide (**37**) as the predominant sesquiterpenes (Figure 10) [65]. Cannabinoids are biologically synthesized from diterpene structures, forming phenol terpenoids, which account for almost 25% of all metabolites [66]. Thus, this diverse terpene profile contributes to the unique aromas of different cannabis “strains” described previously.

Lee et al., 2023 [17], characterized the inflorescences of hybrid cannabis species, i.e., combinations of *C. indica* and *C. sativa*, known as medicinal cannabis, and the terpene compositions in the leaves. It is worth mentioning that the current cultivation and micropropagation of medicinal cannabis, called *Cannabis sativa* L., is carried out by hybridization of seeds/female inflorescences of *Cannabis sativa* and *Cannabis indica* in variable but constant, well-defined percentages (e.g., 50–70% *C. sativa*: 30–50% *C. indica*), which constitute a variety with certification, and genetic lineages are defined and deposited in a database.

The term “terpenoid” is attributed to all terpenes that are naturally or synthetically modified with different functional groups in the hydrocarbon skeleton, particularly hydroxyl groups and methyl groups that may be oxidized or moved at various positions or even removed (Figure 10).

**Figure 11:**
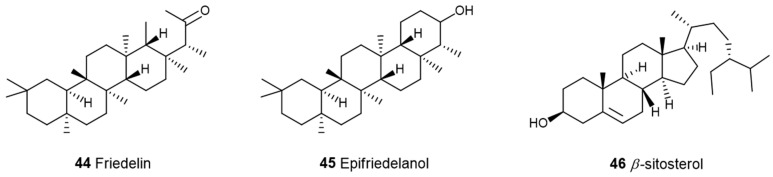
Triterpenoids and a phytosterol found in cannabis.

Terpenoids are well known as bioenhancers, i.e., bioavailability modifiers. Bioavailability refers to the portion of a drug that enters the bloodstream and reaches its intended therapeutic targets. For example, Costa et al., 2015 [67], were the first to assess the ability of triterpenoid to act as a promotor and therefore enhance the permeation of ibuprofen. This triterpenoid, reported by Friedelin (**44**), is also found in cannabis (Figure 11).

Bioenhancers improve bioavailability, thus allowing the drugs to achieve their therapeutic response at lower doses and potentially having the additional benefit of reducing the risk of adverse effects [68]. This concept has been revolutionary in modern medicine [69]. For a bioactive compound to achieve its full therapeutic potential, its bioavailability and absorption are crucial. Factors that can decrease the compound’s bioavailability include the route of administration, with the oral route being the most restrictive, water solubility, permeability limitations, and first-pass metabolism in the liver. Because of their lipophilic chemical nature, such as low water solubility, cannabinoids are prone to poor bioavailability, which is also related to the reported first-pass metabolism of both CBD (**3**) and THC (**2**) [68]. Bioenhancer mechanisms of action address these challenges by, e.g., enhancing absorption, blocking drug efflux membrane transporters, and inhibiting cytochrome P450 (CYP450) liver enzymes.

## 3. Terpenes’ Entourage Effects Studied in Cannabis

Terpenes in cannabis are often referred to as entourage compounds due to their ability to enhance blood–brain barrier permeability, thereby improving the pharmacokinetic properties of, e.g., THC [49,70].

There are over 20,000 different terpenes identified in nature, and they are found in various plants, fruits, and flowers. Terpenes are organic compounds that contribute to the aroma and flavor but also to anti-inflammatory, antimicrobial, and other biologic activities of many plants, including cannabis. Each plant species can produce a unique combination and concentration of terpenes, giving them distinct scents and potential therapeutic properties. In cannabis, terpenes work alongside cannabinoids like THC and CBD to influence the overall effects, potentially playing a role in what is often referred to as the entourage effect.

Case studies follow according to the state-of-the-art identified for studies designed to investigate the potential effect of a cannabis-associated terpene molecule.

### 3.1. β-Caryophyllene and Terpinolene

In a study by A. L. Johnson et al., 2023 [71], terpinolene (TPL) (**15**, Figure 5) had an anxiolytic effect on zebrafish behavior, whereas bisabolol (**40**, Figure 10) had no significant effects. In the second phase of this study, TPL (**15)** and β-caryophyllene (BCP) (**21**, Figure 5) were administered after the administration of rimonabant, AM630, or a control solution. Both TPL (**15**) and BCP (**21**) reduced zebrafish anxiety-like behavior in the open field test when zebrafish was pretreated with the control solution. Zebrafish pretreated with rimonabant did not show any different behavioral responses compared to observations without rimonabant. However, AM630 eliminated the anxiolytic effects of both TPL (**15**) and BCP (**21**). These results indicate that TPL (**15**) and BCP (**21**) have an anxiolytic effect on zebrafish behavior, where CB2R may mediate these effects rather than CB1R. In summary, the study provided direct evidence that CB2R regulates the anxiolytic effects of TPL (**15**) and BCP (**21**). This result confirms the cooperative effect of some terpenes with cannabinoids, which helps to explain the complex effect of cannabis on behavior.

**Figure 12:**
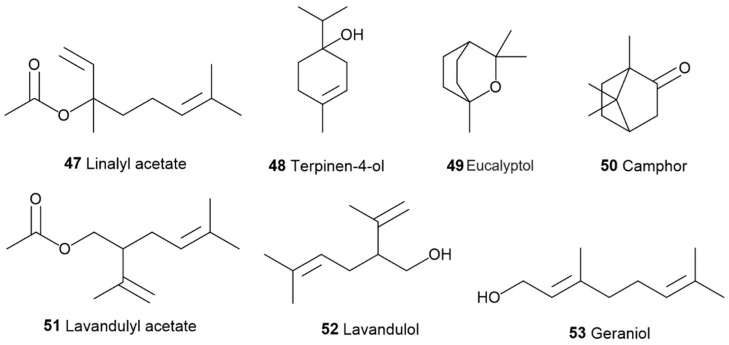
Chemical structures of some of the components in lavender essential oil.

The main components of the lavender essential oil obtained from *Lavandula officinalis* are monoterpene alcohols (60–65%) such as linalool (**20**) (20–50% of the fraction) (Figure 5) and linalyl acetate (**47**) (25–46% of the fraction) (Figure 12). Others include *cis*-ocimene (3–7%) (**16**, Figure 5), terpinen-4-ol (3–5%) (**48**, Figure 12), limonene (**17**, Figure 5), cineole, also known as eucalyptol (**49**, Figure 12), camphor (**50**, Figure 12), lavandulyl acetate (**51**, Figure 12), lavandulol (**52,** Figure 12), *α*-terpineol (**53**, Figure 10), *β*-caryophyllene (**21**, Figure 5), geraniol (**53**, Figure 12), *α*-pinene (**18**, Figure 5), and non-terpenoid aliphatic components [72].

The key terms generally associated include (alone or in combination): lavender, Lavandula, disorder, stress, relaxation, anxiety, sleep, sleeping, and essential oil. Traditionally, essential oil and flowers of *Lavandula officinalis* have been used throughout Europe and worldwide for their sedative properties [73]. Despite several pre-clinical and clinical pharmacology and efficacy studies performed on anxiolytic activity, the EMA (European Medicines Agency) has not yet endorsed lavender flowers and oil for the treatment of general anxiety disorders (cf. ICD-10 F 41.1) [74]. Existing research has highlighted the potential of linalool in treating depression due to its interaction with various targets within the monoaminergic system and its similarity to traditional antidepressant drugs [75]. Linck et al., 2009 [76] found that inhaling linalool at concentrations of 1% and 3% could extend the duration of sleep induced by pentobarbital, lower the body temperature, and slow down the exercise behavior of mice without affecting their coordination. Other studies [77] have shown that linalool (20) can induce sedation, promote relaxation, and decrease aggression and hostility. Additionally, both linalool (**20**) and lavender essential oil have been observed to produce behavioral sedative-like effects by reducing renal sympathetic nerve activity and enhancing parasympathetic nerve activity [78,79]. The modulation of glutamatergic neurotransmission could accomplish the sedative effect of linalool (**20**). Linalool (**20**) has been found, in both in vivo and in vitro studies, to act as a competitive antagonist of the excitatory neurotransmitter glutamate by binding to glutamatergic *N*-methyl-D-aspartate (NMDA) receptors [80]. Furthermore, linalool (**20**) also reduced the release of glutamate triggered by potassium stimulation [81].

No effect has been evidenced so far when linalool is administered with cannabis.

### 3.2. α- and β-Pinenes

The Laws and Smid, 2024 [82], study reveals novel and efficacious neuroprotective and anti-aggregatory effects of *α*-pinene (**18**) and *β*-pinene (**31**) against *β*-amyloid-mediated toxicity. The modest inhibition of lipid peroxidation from *α*-pinene (**18**), *β*-pinene (**31**), and terpinolene (**15**) may also contribute to the multifaceted neuroprotection of *C. sativa*-prevalent terpenes. In addition, limited anti-aggregatory effects were observed for terpineol (**39**), terpinolene (**15**), *α*-pinene (**18**), *β*-pinene (**31**), and friedelin (**44**). The outcomes of this study contribute to an emerging body of knowledge regarding the potential synergistic bioactivities of specific terpenes, which could be valuable in developing medicinal cannabis formulations targeting neurodegenerative diseases.

However, in the above study [82], there is no information concerning the chemical structure characterization of referred compounds. Namely, it is well known that the enantiomers of any bioactive molecule can have distinct physiological and pharmacological activities. One limitation of the research is the absence of information on the structural analysis and purity of the chemical compounds used.

**Figure 13:**
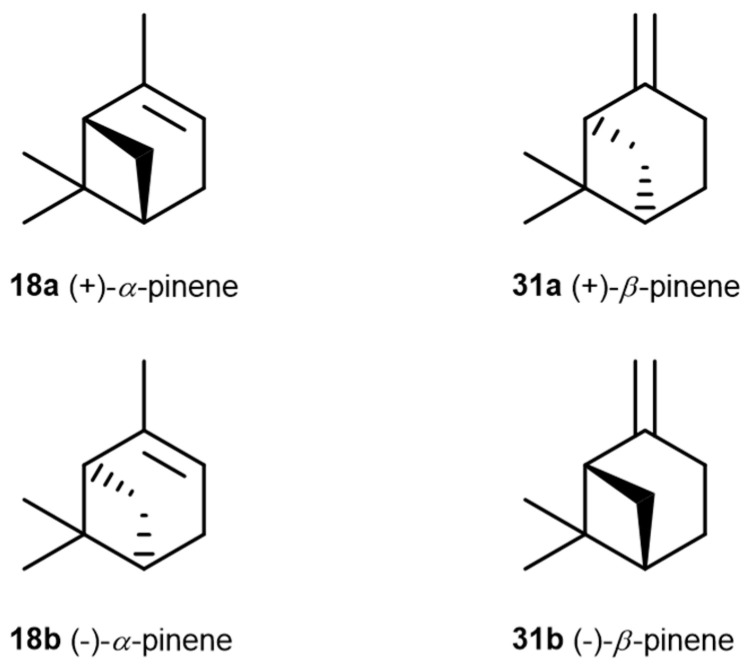
Structural representations of the enantiomers of *α*-pinene and *β*-pinene.

In 2012, there was no clear agreement regarding the antimicrobial properties of pinenes, potentially due to the lack of enantiomer identification [83]. To clarify the controversial results on the subject, Silva et al. [83] conducted a study to assess the antimicrobial effects of the distinct enantiomers and isomers of these monoterpenes (Figure 13). Their research revealed that only the positive enantiomers of pinene exhibited antimicrobial activity against *R. oryzae*, *C. neoformans*, *C. albicans*, and MRSA. The research also highlighted the additive and synergistic effects of (+)-*α*-pinene (**18a**) and (+)-*β*-pinene (**31a**) when combined with commercial antimicrobials, which not only reduced the MIC of combined substances and maintained antimicrobial activity but also lowered toxicity.

### 3.3. β-Myrcene

Local application of myrcene (**14**, Figure 5) at doses of 1 and 5 mg/kg subcutaneously reduced inflammation and joint pain through a cannabinoid receptor-mediated mechanism [84]. The combination of myrcene (**14**) and CBD (**3**) (Figure 1) at 200 μg showed no significant difference in effect compared to myrcene (**14**) alone. Additionally, repeated doses of myrcene (**14**) did not impact joint damage or inflammatory cytokine production. These findings suggest that topical myrcene (**14**) has the potential to ease chronic arthritis pain and inflammation, although it does not exhibit a synergistic effect when combined with CBD (**3**).

### 3.4. Bisabolol, D-Limonene, ⍺-Pinene, and β-Caryophyllene

Recent findings provide a foundation for future research investigating cannabinoid and terpene interactions [85]. Compared to the control, acute administration of bisabolol (**37**) and D-limonene (**17**) increased the food intake, and bisabolol (**40**), D-limonene (**17**), *α*-pinene (**18**), and *β*-caryophyllene (**21**) decreased the time percentage spent in the outer zone in the novelty-induced hypophagia test, suggesting an anxiolytic effect. Social interaction was only improved with ethanol. In contrast to the minor cannabinoids and terpenes, Δ8-THC (**11**) exhibited anxiogenic effects in the marble-burying test after acute administration. During chronic administration, only Δ8-THC (**11**) showed anxiogenic effects in the novelty-induced hypophagia test. Other cannabinoids did not exhibit anxiolytic or anxiogenic effects at the tested doses or times, and neither minor cannabinoids nor terpenes stimulate or impair general motor activity.

### 3.5. β-Caryophyllene, Humulene, Nerolidol, Linalool, and β-Pinene

Blasco-Benito et al., 2018 [15], extracted fresh cannabis flowers with ethanol and then evaporated the solvent, followed by magnetic stirring on a hot plate, thus achieving cannabinoid decarboxylation. The extract (in mg/g) comprised 551.3 THC (**2**), 3.7 CBG (**4**), 3.4 THCA (**5**), and no CBD (**3**), CBC (**6**), CBN (**7**), THCV (**8**), or CBDA (**9**). The five main terpenes were 1.9 *β*-caryophyllene (**21**), 0.6 humulene (**19**), 0.6 linalool (**20**), 0.3 *β*-pinene (**31**), and 0.4 nerolidol (**54**). Although the ethanolic extract of cannabis flowers demonstrated higher antitumor activity than pure THC (**2**), this effect was not attributed to any of the five most prevalent terpenes, as THC (**2**) combined with these five main terpenes did not exhibit higher activity than pure THC (**2**).

## 4. Future Perspectives

With ongoing research into the factors influencing terpene concentrations, biosynthesis, and genetic expression, it may become attainable to develop new cultivars with specific and desired cannabinoids and terpenes [63].

The concept of the entourage effect, first introduced by Ben-Shabat et al. in 1988, describes how the presence of an inactive compound can enhance the activity of an active one, which can be expressed by strict inequality: 1 + 0 > 1 [1].

Synergistic or entourage effects in botanical mixtures or combinations do not necessarily require that the compounds act on the same target to produce an enhanced response. Instead, compounds can exhibit “pharmacodynamic synergism” by interacting with multiple cellular targets, as seen in both antibiotic and cancer synergistic therapies, and/or “pharmacokinetic synergism” by improving the solubility or disposition (absorption, distribution, metabolism) of active constituents, and they can also reduce side effects of the active constituent or disrupt resistance mechanisms [86].

The variety and bioactivities of terpenes found in cannabis, with a focus on minor or secondary terpenes that are present in lower concentrations by mass, can be found in Table S2. The concept of the entourage effect in cannabis and whether the interactions between phytochemicals contribute to a synergetic effect have both been supported and challenged by various studies. The ongoing debate underscores the importance of further research into the interactions between phytochemicals within *Cannabis sativa*, particularly given the growing interest in potential synergy/entourage effects [86].

Meanwhile, systematic databases are essential for gathering experimental evidence on the combinations of major cannabinoids and terpenes contents in cannabis flower and their effects on patient outcomes. Vigil et al., 2023 [87], developed a clinically relevant, user-friendly, and scalable chemovar indexing system that summarizes the primary cannabinoid and terpene profiles and evaluates whether the most consumed chemovars differ in their treatment efficacy and elicited side effects. This chemovar indexing system serves as a proof-of-concept for assessing how distinct phytochemical combinations interact with user-specific characteristics to influence both general and individualized cannabis consumption experiences and health outcomes. Ideally, randomized methods should be employed to evaluate differences in effects across chemovars.

Analysis of the five most frequently consumed chemovars revealed significant differences in effectiveness in symptom treatment for chronic pain, anxiety, and depression (ps < 0.001). While the effects varied in magnitude, all five chemovars were effective for these conditions, except for MC61 (myrcene 0.01–0.49%/*β*-caryophyllene 0.01–0.49%/THC 20–25%/CBD 0.01–1.0%), which exacerbated symptoms of anxiety or depression. Additionally, the chemovars diverged in their association with experiencing positive, negative, and context-specific side effects. Specifically, for two chemovars, MC61 and MC62 (myrcene 0.01–0.49%/*β*-caryophyllene 0.01–0.49%/THC 20–25%/CBD 1–5%), both were associated with two to three fewer positive side effects but up to one more negative and two more context-specific side effects than the other three chemovars.

A hypothesis of a synergistic effect between cannabinoids and terpenes has been postulated given the so-called entourage effect [34]. To date, no reliable scientific evidence of this synergy exists, at least at the cannabinoid receptor level [88]. Nonetheless, it would be premature to deny the existence of either pharmacodynamic or pharmacokinetic interactions among active compounds present in cannabis, as many biological activities have been attributed to its terpenes, including analgesic, anti-inflammatory, and anxiolytic properties [89].

Pioneering works by Fischedick and Hazekamp et al. [90,91] demonstrated already in 2010 and 2012 that terpenes/terpenoids are present in dried medicinal cannabis flower, usually at concentrations in mg/g [90,91]. Also, as foreseen by Casano et al., 2011 [92], “the relative content of terpenoids is strongly inherited while total yield per weight of tissue is more subjected to environmental factors”. The relative content (%) of terpenes and terpenoids is commonly employed for chemo-systematic studies, as demonstrated in this publication. Since receptors typically require their ligands at very low concentrations, understanding these terpenes and terpenoids could be important for therapeutic treatments. Thus, any preclinical studies and clinical trials using cannabis-herbal preparations and/or extracts, purified fractions, or any other product with medical purposes shall perform an exhaustive analytical characterization of all compounds that are present in the herbal-derived product, even in very low concentrations, which are not being currently considered [93].

The observations discussed above have led to the proposal that THC (**2**) might be considered a “silver bullet” in therapeutic contexts, while other compounds derived from cannabis could function collectively as a “synergistic shotgun” [33,94].

Until now, the majority of studies on synergy have concentrated mainly on the interactions between cannabinoid structures, although the original definition of the entourage effect arose from the interaction between 2-acyl-glycerol esters and cannabinoids. The potential for synergic effects from volatile and easily metabolized monoterpenes and monoterpenoids is limited by their very low concentrations in many cannabis preparations and their mode of administration. Oral consumption typically results in minimal therapeutic levels due to extensive first-pass metabolism. Even inhalation, which might be expected to provide the best chance for these terpenes to have an impact, is also constrained by the high rates of clearance and short half-lives of some monoterpenes, suggesting that accumulation to therapeutic levels is unlikely [95]. Moreover, polyphenolic compounds, such as flavonoids, exhibited poor oral bioavailability, leading to very low plasma concentrations in vivo after ingestion [96]. Consequently, the role of polyphenolic constituents of cannabis in contributing to any proposed entourage effect appears to be very limited.

Linking to the current market lines largely described in general internet sites in the area of cannabis products and as described in Section 5 of this article, search motifs and evidence-based medicine will have to follow the classic paths with preclinical and clinical studies of chemically well-characterized cannabis-based preparations to validate the truly influential effects of terpenes when administered jointly with cannabis, whether orally, with or without vaporization, or by inhalation. Table 1 summarizes the state-of-the-art of known individual effects of terpenes that can characterize them as influencers for potentially observable final effects.

## 5. Methodology

To achieve the objectives, a review of the scientific literature on cannabis entourage effects was designed, and the PRISMA model (Preferred Reporting Items for Systematic Reviews and Meta-Analyses) was used to organize the information. Two research questions were formulated: (1) What are the physiological effects of terpenes and terpenoids found in cannabis? (2) What are the proven entourage effects of terpenes in cannabis?

The first methodological approach to identify publications was an exploratory search in electronic databases with predefined keywords. Subsequently, the most relevant articles were consulted, key phrases and search terms were identified, and Boolean phrases were defined for a systematic final search. The respective descriptors were identified using the medical subject headings (MeSH) terms in the PubMed/MEDLINE, Web of Science, and EBSCO databases (Library, Information Science & Technology Abstracts, and Academic Search Complete).

An electronic bibliographic search was carried out using MEDLINE via PubMed, Scopus, Web of Science, and Cochrane, covering the total accessible period until 22 December 2023, with the following questions:

(1) What are the physiological effects of terpenes and terpenoids found in cannabis?
  (((Physiological Effects) AND (Terpenes)) AND (Terpenoids)) AND (Cannabis);
  366 results, only 13 answered objectively to the question.
(2) What are the proven entourage effects of terpenes in cannabis?
  ((Entourage Effects) AND (Terpenes)) AND (Cannabis);
  49 results, only 16 answering objectively to the question.

To obtain the most accurate information possible, we additionally searched each terpene considered most representative and analyzed each article to find concrete studies of the entourage effect in cannabis. The results are summarized in Table 2, which reports how 1424 were found and duplicates were eliminated.

All articles located through various scientific repository platforms (B-on, Scopus, Web of Science, Pub Med) were analyzed.

**Figure 14:**
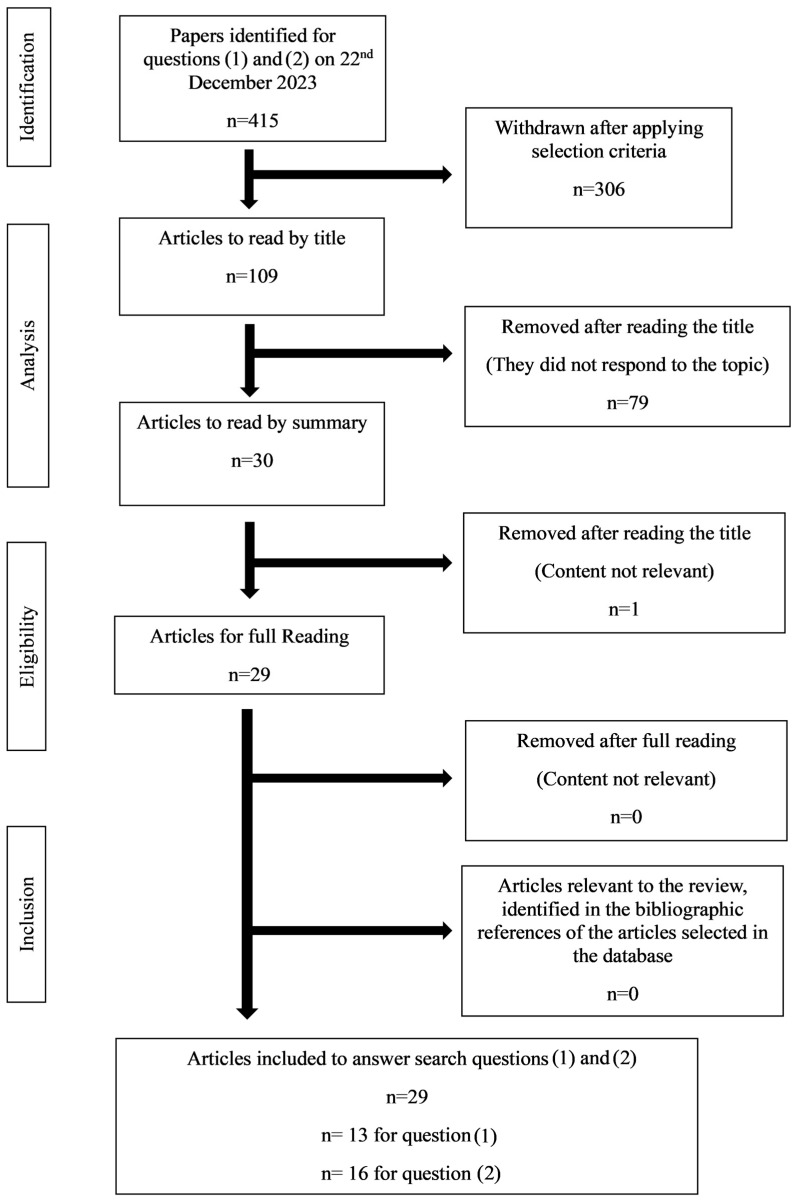
Prisma flow diagram of the research results for questions (1) and (2).

Of the 415 articles relating to the main research questions, (1) and (2), formulated, all assessed these one by one and were considered enlightening to answer the question. Thus, 29 articles are cited in this document (Figure 14), although 113 in total are referenced to understand the background and the general information under the state-of-the-art of the evidence-based cannabis entourage effect.

## 6. Conclusions

The term entourage effect is frequently employed in the field of medicinal cannabis to describe a type of herbal synergy, wherein the primary cannabinoids, Δ9-THC (**2**) and CBD (**3**), are thought to have their effects enhanced or modulated by other compounds present in the plant and its extracts. This concept is plausible, particularly when considering minor phytocannabinoids, monoterpenes, sesquiterpenes, and sesquiterpenoids. However, the practical application of this effect is complicated by several factors, including variability in the levels of minor secondary metabolites, across different cannabis preparations, the often-limited scope of analytical methods used, and the low bioavailability of many of these components of interest.

A major challenge in leveraging the entourage effect clinically is akin to issues faced by many herbal medicines; without a clear understanding of the key active agents, it is very difficult to produce reliable products with consistent levels of these constituents. For cannabis, even products with consistent levels of the same chemovar can exhibit variability in their secondary metabolite profiles due to differences in cultivation conditions and processing methods. While employing a combination of HPLC and GC for profiling may improve product consistency, this approach relies on the assumption that all the significant entourage compounds can be detected using these techniques. Further, to the author’s knowledge, there are no clinical trials specifically designed to validate the entourage effect in medicinal cannabis.

In conclusion, while current research suggests a potential overlap in therapeutic benefits between cannabinoids and terpenes as influencers, the hypothesis that these effects are additive or synergistic remains unproven. Further research is expected to understand which factors may enhance cannabinoid efficacy in an additive or synergistic manner.

## Figures and Tables

**Table 1:** The prevalent cannabis terpenes as influencers aligned with business market attributes.

Terpene	Potential Effect	Type of Evidence
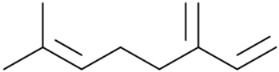 Myrcene (**14**) 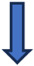 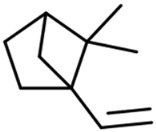 Hashishene (**109**)	Relaxation	*Cannabis sativa* is known to contain *β*-myrcene (29.4–65.8%) of the steam-distilled essential oil from various fiber and drug strains tested in modern cannabis cultivars within North America [97]. When administered orally, a single dose of *β*-myrcene has been shown to extend the duration of pentobarbital-induced sleep when administered 60 min before a barbiturate [98]. Additionally, *β*-myrcene undergoes photo-oxidation to form “hashishene”, a compound notable for its high concentration in hashish [99].
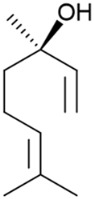 Linalool (**20**)	Anxiolytic and antidepressant	Linalool, a major compound of lavender essential oil, is traditionally used and has been approved by the EMA as an herbal medicinal product for alleviating mild symptoms of mental stress and exhaustion and aiding sleep [74]. Some animal and clinical studies have shown positive outcomes in models of anxiety and depression; however, research into the molecular mechanisms underlying these effects remains limited [100].
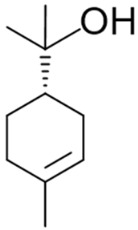 Terpineol (**39**)	Uplifting	Terpineol exists in four isomer forms: *α*-, *β*-, and γ-terpineol and terpinen-4-ol. 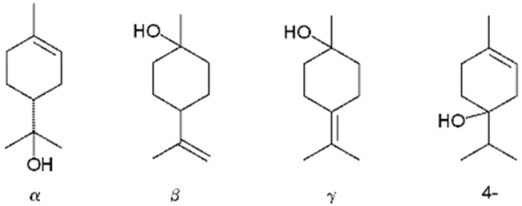 *β*- and γ-terpineol differ only in the position of the double bond. Typically, terpineol is a mixture of these isomers, with *α*-terpineol being the most prevalent. Terpineol is noted for its anticancer, anticonvulsant, antihypertensive, antioxidant, antinociceptive, and antiulcer effects [101].
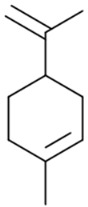 Limonene (**17**)	Stress relief	D-limonene has demonstrated protective effects against the nephrotoxic side effects of the anticancer drug doxorubicin (Dox) [102].
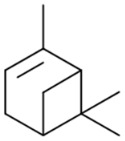 *α*-Pinene (**18**)	Soothing	*α*-Pinene (60% human pulmonary bioavailability) is an anti-inflammatory and an acetylcholinesterase inhibitor, aiding memory [33]. It also interacts with the benzodiazepine binding site [103]. However, the hypothesis that *α*-pinene may mitigate memory deficits associated with THC consumption due to its acetylcholinesterase inhibition remains unproven. However, ongoing studies propose that potential role as an influencer.
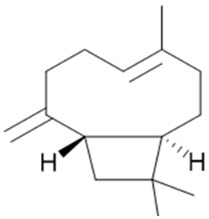 Caryophyllene (**21**)	Pain relief 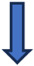 Anti-inflammatory action, caryophyllene improves cold tolerance and acts as a potential adjuvant for human colorectal cell growth inhibition	*β*-Caryophyllene acts as a full agonist of cannabinoid receptor type 2 in rats with a binding affinity of K_i_ = 155 nM. [104] and exhibits anti-inflammatory effects. In comparison, cannabinol (CBN) binds to CB_2_ receptors as a partial agonist with an affinity of K_i_ = 126.4 Nm [105], and THC binds as a partial agonist with an affinity of K_i_ = 36 nM [106]. *β*-Caryophyllene has been shown to enhance cold tolerance at low ambient temperatures. For example, wild giant pandas frequently use *β*-caryophyllene and caryophyllene oxide found in horse manure to inhibit transient receptor potential melastatin 8 (TRPM8), an archetypical cold-activated ion channel in mammals [107]. Additionally, in an in vitro human colorectal adenocarcinoma study, a combination of *β*-caryophyllene (10 μg/mL) and paclitaxel (0.025 μg/mL) resulted in greater inhibition of cancer cell growth than paclitaxel used alone [108].
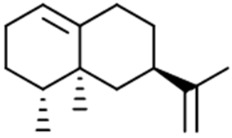 Valencene (**108**)	Protection of cartilage and alleviation of the progression of osteoarthritis	Valence demonstrates protective effects on cartilage and alleviation of the progression of osteoarthritis by anti-inflammatory anti-oxidative stress effects [109].
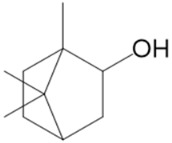 Borneol (**60**)	“Sedative” 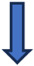 Anticonvulsant and antinociceptive properties	Borneol, known for its ability to cross the blood–brain barrier, modulates GABAergic activity in the central nervous system, exhibiting anticonvulsant and antinociceptive properties [110].
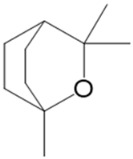 Eucalyptol (**49**)	“Relaxation” 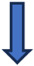 Relief of symptoms of localized muscle pain	Eucalyptol, comprising approximately 70–90% of eucalyptus oil, has been endorsed by the Committee on Herbal Medicinal Products (HMPC) of the EMA for its long-standing use in reliving cough associated with the common cold and localized muscle pain [111].

**Table 2:** Search keywords and results in the PubMed Platform for the period 2013–2023.

Terpene	Search Keywords	N. Publications
Myrcene (relaxing)	(myrcene) AND (cannabis)	58 None for relaxation 1 showing no effect
Linalool (sedative)	(linalool) AND (cannabis)	6 31 for sedative effects alone
Terpinolene (uplifting)	(terpinolene) AND (cannabis)	1133 Terpinolene x effect: no uplifting-related studies
Limonene (stress relief)	(limonene) AND (cannabis)	42 3 for stress relief
Pinene (soothing)	(pinene) AND (cannabis)	48 0 for soothing
Caryophyllene (pain relief)	(caryophyllene) AND (cannabis)	95 5 for analgesic synergies/cannabis terpenes and CBD
Valencene (euphoria)	(valencene) AND (cannabis)	0—no study on cannabis
Borneol (sedative)	(borneol) AND (cannabis)	2 with cannabis 10 references for borneol AND as a sedative
Eucalyptol (relaxing)	(eucalyptol) AND (cannabis)	6~3/5 on eucalyptol AND relaxation but in mixtures

## Supplementary Materials

The following supporting information can be downloaded at https://www.mdpi.com/1424-8247/17/11/1543/s1: Table S1: Selected *Cannabis sativa* L.-derived cannabinoids, their targets, mechanisms of action, and potential resultant pharmacological effects; Table S2: Selected *Cannabis sativa* L.-derived terpenes, their targets, mechanisms of action, and potential resultant pharmacological effects.
