# Supplementary material for: The Entourage Effect in Cannabis Medicinal Products: A Comprehensive Review
Source: Pharmaceuticals (Basel). 2024 Nov 17;17(11):1543. doi: 10.3390/ph17111543 (PMC11870048; doi:10.3390/ph17111543)
Supplement: Supplementary file 1 [file pharmaceuticals-17-01543-s001.zip › pharmaceuticals-3231074-supplementary.pdf]

Table S1. Selected *Cannabis sativa* L.-derived cannabinoids, their targets, mechanisms of action, and potential resultant pharmacological effects – Table adapted from (Christensen et al., 2023)

**Structures**	**Targets**	**Mechanisms of Action**	**Potential Pharmacological effects**
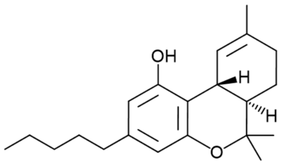 **Δ9-Tetrahydrocannabinol (THC) (2)**	CB1	Partial agonist	Analgesic **, ***<br>Anti-convulsant **<br>Anti-epileptic **<br>Sleep improvement **, ***<br>Anti-anorectic **, ***<br>appetite stimulating **, ***<br>Anti-emetic **, ***<br>Anxiolytic **
	CB2	Partial agonist	Analgesic **, ***
	GPR55	Agonist	Not reported
	GPR18	Agonist	Not reported
	5-HT-3A	Antagonist	Anti-nociception *<br>Anti- emetic *
	DOR	Negative allosteric modulator	Not reported
	MOR	Negative allosteric modulator	Not reported
	PPAR-y	Agonist	Anti-cancer, anti-proliferative *, **
	GlyR	Agonist	Analgesic *, **
	TRPV2	Agonist	Not reported
	TRPV3	Agonist	Not reported
	TRPV4	Agonist	Not reported
	TRPA1	Agonist	Not reported
	TRPM8	Antagonist	Not reported

**Cannabidiol (CBD) (3)**<br>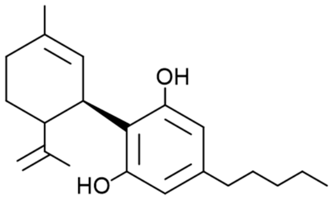<br>**Cannabidiol (CBD) (3)**	CB1	Negative allosteric modulator<br>Antagonist	THC-related adverse effects modulation **, ***<br>Anxiolytic **<br>Antidepressant **<br>Vasorelaxant **
	CB2	Partial agonist<br>Negative allosteric modulator<br>Antagonist	Seizure reduction **<br>Anti-epileptic **<br>Anti-inflammatory **<br>Anti-cancer *, **<br>Body weight decrease **<br>Neuroprotection **
	GPR3	Inverse agonist	Alzheimer’s disease improvement *
	GPR6	Inverse agonist	Parkinson’s disease improvement *
	GPR12	Inverse agonist	Anti-cancer *
	GPR55	Antagonist	Anti-epileptic **, ***<br>Seizure dampening **<br>Bone resorption inhibition **<br>Parkinson’s motor skills improvement **<br>Cancer cell migration inhibition
	FAAH	Inhibitor	AEA increase and related effects *<br>Sleep induction *, **<br>Stress reduction ***<br>Anxiolytic ***<br>Anti-depressant **
	5-HT-1A	Agonist<br>Inverse agonist	Anti-emetic *, **<br>Analgesic **

			Chemotherapy induced neuropathic pain reduction *, **<br>Anxiolytic **<br>Anti-depressant **<br>Cognitive performance improvement **<br>Anti-epileptic *, **, ***<br>Seizure reduction **<br>Anti-stress **<br>Neuroprotection **
	5-HT-3A	Antagonist	Anti-emetic **<br>Cardiovascular effects **
	A1A	Agonist	Anti-arrhythmic **<br>Analgesic **
	A2A	Agonist	Anti-inflammatory *, **<br>Cognitive performance improvement **
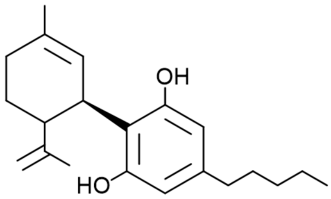<br>**Cannabidiol (CBD) (3)**	PPAR-γ	Agonist	β-amyloid-induced neuroinflammation reduction *, **<br>Hippocampal neurogenesis *, **<br>Alzheimer's disease improvement *, **
	Immune cell (not further specified)	Inhibitor<br>Activator	Anti-inflammatory *, **<br>Immunosuppressive *, **<br>Cytokine release reduction/increase *, **<br>Anti-arthritic **<br>Multiple sclerosis amelioration **
	Gly-$\alpha 1$	Positive allosteric modulator	Anti-inflammatory *<br>Neuroprotective *

		Agonist	
	Gly-α3	Positive allosteric modulator	Analgesic **
	GABA-A	Positive allosteric modulator	Anti-convulsant **<br>Anti-epileptic **
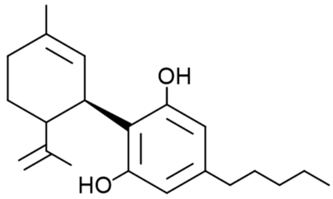<br>**Cannabidiol (CBD) (3)**	TRPV1	Agonist	Neuron anti-hyperexcitability *<br>Anxiolytic **<br>Anti-cancer, apoptosis *<br>Microglial phagocytosis enhancement *<br>Cardiovascular effects **
	TRPV2	Agonist	Microglial phagocytosis enhancement *
	TRPV3	Agonist	Not report
	TRPV4	Agonist	Not report
	TRPA1	Agonist	Analgesic **
	TRPM8	Antagonist	Not reported
	DOR	Negative allosteric modulator	Not reported
	MOR	Negative allosteric modulator	Not reported
	D2	Partial agonist	Anti-psychotic*
	CB2	Partial agonist	Anti-inflammatory *, **<br>Colitis attenuation *, **
	AEA uptake	Inhibitory	Various effects related to AEA *
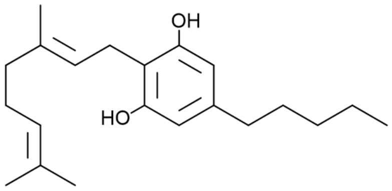<br>**Cannabigerol (CBG) (4)**	5-HT-1A	Antagonist	Reverse anti-emetic effect of, *e.g.* CBD **
	A2A	Agonist	Not reported
	TRPV1	Agonist	Not reported

	TRPA1	Agonist	Not reported
	TRPM8	Antagonist	Colon anti-cancer **
**Δ9-Tetrahydrocannabinolic acid (THCA) (5)** 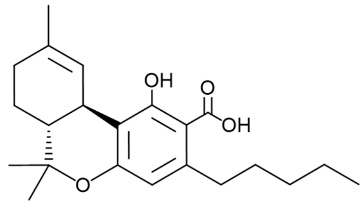 **Δ9-Tetrahydrocannabinolic acid (THCA) (5)**	CB1	Partial agonist	Anti-nociceptive ** Anti-inflammatory
	CB2	Agonist	Not reported
	PPAR-γ	Agonist	Adiposity reduction ** Metabolic syndrome prevention ** Anti-inflammatory ** Neuroprotective *, **
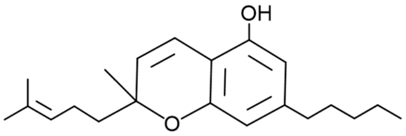 **Cannabichromene (CBC) (6)**	CB2	Agonist	Anti-inflammatory *
	AEA uptake	Inhibitor	Various effects related to AEA *
	TRPV3	Agonist	Not reported
	TRPV4	Agonist	Not reported
	TRPA1	Agonist	Anti-inflamatory ** Colitis reduction ** Analgesic **
	TRPM8	Antagonist	Not reported
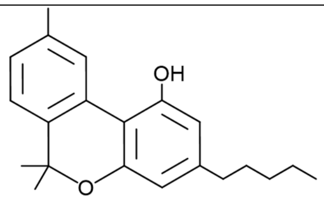 **Cannabinol (CBN) (7)**	CB1	Agonist	Appetite increase **
	CB2	Agonist Inverse agonist	Not reported
	TRPA1	Agonist	Not reported
	TRPM8	Antagonist	Not reported
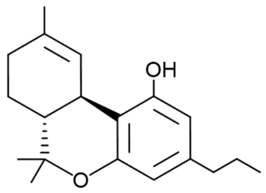 **Δ8-Tetrahydrocannabivarin (THCV) (8)**	CB1	Agonist Antagonist	Anti-psychoactive (*e.g.* reverse THC-induced psychoactive effects) ** Analgesic ** Anti-convulsant ** Anti-epileptic * Hypophagia and weight reduction ** Glycemic control improvement **, ***

	CB2	Partial agonist<br>Antagonist	Anti-inflammatory **<br>Inflammatory pain reduction **
	5-HT-1A	Agonist	Antipsychotic *, **
	TRPV2	Agonist	Not reported
	TRPA1	Agonist	Not reported
**Δ8-Tetrahydrocannabivarin (THCV) (8)**	TRPM8	Antagonist	Not reported
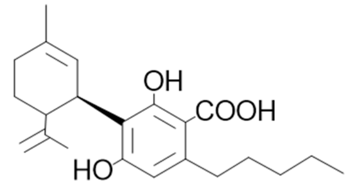<br>**Cannabidiolic acid (CBDA) (9)**	CB2	Partial agonist	Not reported
	5-HT-1A	Agonist	Anti-emetic **<br>Anti-convulsant **<br>Anxiolytic **
	TRPV1	Agonist	Anti-heperalgesic **
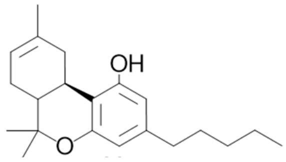<br>**Δ8-Tetrahydrocannabinol (THC) (11)**	CB1	Partial agonist	Appetite stimulant **
	CB2	Agonist	Not reported
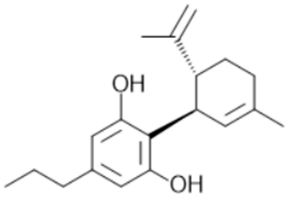<br>**Cannabivarin (CBDV) (24)**	GABA-A	Positive allosteric modulator	Anticonvulsive *, ***<br>Anti-epileptic *, ***
	TRPV1	Agonist	Neuronal anti-heperexcitability *<br>Anti-convulsant **
	TRPV2	Agonist	Not reported
	TRPV3	Agonist	Not reported
	TRPA1	Agonist	Not reported

*: Pre-clinical *in vitro* study; **: pre-clinical *in vivo* study; ***: clinical study; N.B.: This table is non-exhaustive, broadly elucidating selected compounds and some of their potential pharmacological effects currently present in the pre-clinical literature. Depending on study parameters, the compounds show differing, sometimes biphasic, affinities and effects at different targets, thus highlighting the contradictory and equivocal evidence state. **Abbreviations:** 5-hydroxytryptamine receptor 1A (5-HT-1A); 5-hydroxytryptamine receptor 3A (5-HT-3A); adrenergic receptor alpha-1 (A1A); adrenergic receptor alpha-2 (A2A); anandamide endocannabinoid (AEA); cannabinoid receptor 1 (CB1); cannabinoid receptor 2

(CB2); delta-opioid receptor (DOR); dopamine D2 receptor (D2); fatty acid amide hydrolase enzyme (FAAH); gamma-aminobutyric acid type A receptor (GABA-A); glycine receptor (GlyR); glycine receptor type $\alpha 1$ (GlyR-$\alpha 1$); glycine receptor type α 3 (GlyR-$\alpha 3$); G-protein-coupled receptor 2 (GPR2); Gprotein-coupled receptor 3 (GPR3); G-protein-coupled receptor 6 (GPR6); G-protein-coupled receptor 12 (GPR12); G-protein-coupled receptor 18 (GPR18); G-protein-coupled receptor 55 (GPR55); Mu-opioid receptor (MOR); peroxisome proliferator-activated receptor gamma (PPAR-γ); transient receptor potential cation channel type A1 (TRPA1); transient receptor potential cation channel 8 (TRPM8); transient receptor potential vanilloid type 1 (TRPV1); transient receptor potential vanilloid type 2 (TRPV2); transient receptor potential vanilloid type 3 (TRPV3); transient receptor potential vanilloid type 4 (TRPV4).

Table S2. Selected *Cannabis sativa* L.-derived terpenes, their targets, mechanisms of action, and potential resultant pharmacological effects. Table adapted from (Christensen et al., 2023)

Structures	Targets	Mechanisms of Action	Potential Pharmacological effects
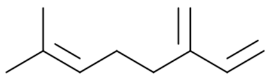 **Myrcene (14)**	TRPV1	Agonist	Analgesic *
	A2A	Agonist	Analgesic **
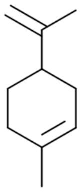 **Limonene (17)**	5-HT-1A	Agonist	Anti-stress ** Anxiolytic ** Anti-depressant **
	TRPA1	Agonist	Analgesic **
	NFκB	Inhibitor	Anti-inflammatory **, *** Analgesic ** Colitis reduction **
	A2A	Agonist	Not reported
	FTase	Inhibitor	Anti-cancer **
	MAPK NFκB	Inhibitor	Anti-inflammatory **
	ERK/AKT	Agonist	Anti-cancer *, **
	Virus particle (not further specified)	Inhibitor	Anti-viral *
	A1A	Agonist	Analgesic **
	A2A	Agonist	Analgesic **
	GABA-A	Agonist	Anxiolytic **

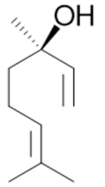 **Linalool (20)**	Cancer cell (not further specified)	Inhibitor	Anti-cancer *, **
**Caryophyllene (21)**	CB2	Agonist	Analgesic **<br>Chemotherapy-induced peripheral neuropathy attenuation **<br>Anti-inflammatory**<br>Steatohepatitis protecting **<br>Metabolic dysregulation attenuation **
	PPAR-α	Agonist	Intracellular lipid modification*<br>Steatohepatitis protecting*
	PPAR-γ	Agonist	Intracellular lipid modification*<br>Steatohepatitis protecting*
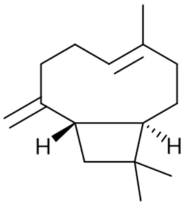	MAPK	Inhibitor<br>Agonist	Chemotherapy-induced peripheral neuropathy attenuation **<br>Anti-cancer *
**Caryophyllene (21)**	TLR4	Inhibitor	Microglial activation inhibition **<br>Neuroprotective *, **<br>Anti-inflammatory *, **

* Pre-clinical *in vitro* study. ** Pre-clinical *in vivo* study. *** Clinical study. N.B.: This table is non-exhaustive, broadly elucidating selected compounds and some of their potential pharmacological effects currently present in the pre-clinical literature. Depending on study parameters, the compounds show differing, sometimes biphasic, affinities and effects at different targets, thus highlighting the contradictory and equivocal evidence state. **Abbreviations**: 5-hydroxytryptamine receptor 1A (5-HT-1A); adrenergic receptor alpha-1 (A1A); adrenergic receptor alpha- 2 (A2A); cannabinoid receptor 2 (CB2); Extracellular-regulated kinase/serine/threonine kinase (ERK/AKT); farnesyltransferase (FTase); gamma-aminobutyric acid type A receptor(GABA-A); mitogen-activated protein kinase (MAPK); Nuclear factor kappa B (NFκB); peroxisome proliferator-activated receptor alpha/gamma (PPAR- α/γ); Toll-like receptor 4 (TLR4); transient receptor potential cation channel type A1 (TRPA1); transient receptor potential vanilloid type 1 (TRPV1).
